# Myopic traction maculopathy biomarkers on optical coherence tomography angiography—An overlooked mechanism of visual acuity correction in myopic eyes

**DOI:** 10.1038/s41433-019-0424-0

**Published:** 2019-04-01

**Authors:** Shih-Wen Wang, Kuo-Chi Hung, Chia-Ying Tsai, Muh-Shy Chen, Tzyy-Chang Ho

**Affiliations:** 1 Department of Ophthalmology, Shuang Ho Hospital, Taipei Medical University, No. 291, Zhongzheng Rd, Zhonghe District New Taipei City, 23561 Taiwan; 2 Department of Ophthalmology, National Taiwan University Hospital, College of Medicine, National Taiwan University, No. 7, Chung-Shan S. Rd, Taipei City, 10002 Taiwan; 3 Sinying Hospital, Ministry of Health and Welfare, Department of Ophthalmology, No. 73, Xinyi St., Xinying Dist. Tainan City, 73042 Taiwan; 4 Department of Ophthalmology, Fu Jen Catholic University Hospital, No. 362, Zhongzheng Road, Xindian Dist. New Taipei City, 23148 Taiwan; 5 School of Medicine, College of Medicine, Fu-Jen Catholic University, New Taipei City, Taiwan; 6 Department of Ophthalmology, Cardinal Tien Hospital, Fu Jen Catholic University, No.362, Zhongzheng Rd, Xindian Dist. New Taipei City, 23148 Taiwan

**Keywords:** Biomarkers, Predictive markers

## Abstract

**Background/objectives:**

Myopic traction maculopathy of the retinoschisis type is a unique entity that has been frequently overlooked in refraction correction in daily practice in myopic eyes. The objective of this study was to describe the imaging characteristics of myopic traction maculopathy (MTM) seen on optical coherence tomography angiography (OCTA) and to detect biomarkers of the associated functional changes.

**Subjects/methods:**

We performed OCTA on eyes with MTM and retinoschisis (RS group; *n* = 27) and highly myopic eyes without MTM (control group; *n* = 27). The RS group was further divided into a dome-shaped macula (DSM) group and a non-DSM group. The groups were compared for morphologic and perfusion characteristics.

**Results:**

The RS and control groups had comparable spherical equivalence (*p* = 0.65), but the RS group had worse best-corrected visual acuity (*p* < 0.01), larger retinal volumes (*p* < 0.01), and greater subfoveal choroid capillary vessel density values (*p* = 0.04). Compared to the non-DSM group, the DSM group had significantly smaller inner retinal volumes (*p* = 0.05) and significantly larger choroid capillary vessel density values in both the whole macula (*p* = 0.005) and the fovea (*p* = 0.03).

**Conclusions:**

Our high-resolution OCTA morphologic and vascular perfusion data correlated well with functional abnormalities encountered in myopic eyes. Changes in vessel density may elucidate the pathogenesis of MTM and characterize the mechanical stretch forces acting in eyes with MTM.

## Introduction

Myopic traction maculopathy of the retinoschisis type is a unique entity that has no obvious anatomical abnormalities, except for foveoschisis of the sensory retina. The condition is frequently overlooked in the refraction correction of visual acuity in daily practice in myopic eyes. The term “myopic foveoschisis” was coined by Takano and Kishi to describe retinal splitting at the macula observed on optical coherence tomography (OCT) in patients with high myopia and posterior staphyloma [1]. In 2004, Panozzo et al. proposed the term “myopic traction maculopathy” (MTM) to describe the macular damage caused by traction forces from the epiretinal membrane or residual focal vitreoretinal adhesion combined with posterior staphyloma and progressive scleral stretching in myopic eyes [2]. OCT is essential when evaluating highly myopic eyes, in which MTM is suspected because it can provide detailed images of macular damage and may also elucidate the poorly understood pathogenesis of MTM. The incidence of myopia has increased in recent decades in many countries, particularly East Asia [3, 4], so managing its comorbidities is becoming increasingly important.

One study suggested that MTM manifests as three sometimes overlapping subtypes, i.e., retinoschisis, foveal detachment, and macular holes [5]. The impairments in visual acuity associated with each subtype have been described previously [5, 6] When foveal detachment or macular holes occur, patients usually require surgical intervention. Many patients with retinoschisis experience slow, progressive visual deterioration, although some may have stable visual acuity for years, even with retinal thickening, appearance of foveal cysts, or retinal detachment [7]. Gaucher et al. proposed the presence of pre-macular structures as a predisposing factor for future visual deterioration in a retrospective observational case series study of patients with MTM [8].

Benhamou et al. examined eyes with retinoschisis in a retrospective observational case series study and found that all exhibited outer retinoschisis and that 29% also showed inner retinoschisis [7]. Additionally, Shimada et al. found that progression of MTM was associated with an increased height or extent of macular retinoschisis and development of macular foveal retinal detachment [9]. However, the mechanism underlying visual deterioration in MTM-affected eyes, exclusively exhibiting retinoschisis, remains unknown.

Dome-shaped macula (DSM), a novel pathology in myopic maculopathy, was first reported by Gaucher et al. [10]. Using OCT, they observed an inward bulge within the myopic staphyloma that was accompanied by visual impairment. Swept-source OCT and three-dimensional reconstruction revealed that the dome shape was caused by localized thickening of the macular sclera [11]. The incidence of foveoschisis is lower in patients with MTM and DSM than in those who have MTM without DSM. The scleral bulge is thought to reflect a macular buckling effect that prevents foveoschisis.

OCT angiography (OCTA) can be used to evaluate the vasculature and perfusion of the retina and choroid. These technologies have been used in eyes with diabetic retinopathy, polypoidal choroidal vasculature, and juvenile foveoschisis with promising [12–15].

In this study, we used these imaging techniques to compare the imaging features of highly myopic eyes with and without MTM and those in eyes with MTM that did and did not have a DSM. We limited our focus to MTM of the retinoschisis subtype without foveal detachment or macular holes in order to investigate the pathogenesis of visual impairment within that specific subtype.

## Subjects and methods

The study was approved by the ethics committee at National Taiwan University Hospital and was performed in accordance with the principles of the Declaration of Helsinki. All patients who participated in the study gave written informed consent after receiving an explanation of the nature of the study and possible consequences. Patients with high myopia who underwent OCTA at the high myopia clinic in the hospital’s ophthalmology department between January 1, 2017 and June 30, 2017 were eligible for inclusion in the study. High myopia was defined as a spherical equivalence of < −6.0 diopters or an axial length of > 26.5 mm [16]. Patients who had MTM with retinoschisis (RS), defined as retinal splitting within a central square measuring 3 mm × 3 mm in the OCTA images, were allocated to an RS group (*n* = 27) and those without MTM or other structural deterioration served as a control group (*n* = 27). The exclusion criteria were lacquer cracks, choroidal neovascularization, macular atrophy, MTM of the macular hole or foveal detachment subtype, previous vitreoretinal surgery or intravitreal injections, and diabetes mellitus.

Data on age, sex, most recent axial length measurement, and whether the eyes were phakic or pseudophakic were recorded for each patient. Refraction data were used to calculate each patient’s best-corrected visual acuity (BCVA) as the logarithm of the minimum angle of resolution (logMAR).

We documented the presence of DSM, which was confirmed with 10-mm cross line scans. Subfoveal choroidal thickness was measured manually from the outer surface of the hyper-reflective line ascribed to the retinal pigment epithelium (RPE) to the hyper-reflective line of the inner scleral border at the foveal center [16]. The integrity of the ellipsoid zone was also recorded.

### Image acquisition and analysis

The OCTA images were acquired using an Avanti RTVue XR device with the AngioVue system (Optovue, www.optovue.com). This system can reach an acquisition rate of 70,000 a-scans per second, achieving high axial resolution at depths of up to 5 μm and minimizing motion artifact. Each OCTA cube scan consisted of 304 × 304 a-scans within a 3-mm × 3-mm square centered on the fovea, which yielded 304 b-scans. Each b-scan output displayed the average of at least two individual scans. The AngioVue software includes a built-in projection artifact removal algorithm and has four default en face retinal imaging settings for automatic segmentation of the superficial layer (SL), deep layer (DL), outer retina, and choriocapillaris. The SL was outlined with an inner boundary 3μm below the internal limiting membrane (ILM) and an outer boundary 15μm below the inner plexiform layer. The DL was segmented with boundaries set at 15μm and 70μm below the inner plexiform layer. The boundaries for the choriocapillaris were defined as 30;μm and 60;μm below the RPE. The precision of automated segmentation was checked by reviewing each of the 304 b-scans in every OCTA cube scan. Auto-segmentation errors were corrected by manually adjusting the contour and location of each segmentation line. The imaging data were interpreted by two independent readers. Disagreements were resolved by discussion between the two readers and another senior retinal specialist. The quality of the OCTA images was assessed using the signal strength index.

We evaluated the foveal avascular zone (FAZ) in the SL and in the DL by analyzing en face images saved as PNG files in the AngioVue system using ImageJ (National Institutes of Health, Bethesda, MD, USA; http://rsb.info.nih.gov/ij/index.html). To facilitate the measurements, each FAZ area was outlined twice manually following conversion to binary images. Projection artifacts from the SL were excluded when outlining the FAZ in the DL. FAZ areas were calculated (in mm^2^) by multiplying the number of pixels by (3/304)^2^mm^2^/pixel. A built-in tool in the AngioVue system was used to measure the choroidal flow area (i.e., the area of flow within a circle with a diameter of 2mm centered over the choriocapillaris slab in en face images) and vessel density in the SL, DL, and choriocapillaris [17, 18]. We defined vessel density as the percentage of the cross-sectional area of the vessel occupied by the lumen. We defined whole-macula density and foveal vessel density as the density value within a 3-mm × 3-mm square and a 1-mm-diameter circle, respectively. The retinal volume was calculated as the sum of the retinal thickness values, which were mapped onto a 5-mm × 5-mm square grid centered on the fixation point with grid spacings of 0.25 mm in the inner 4-mm × 4-mm area and 0.5 mm in the outer area. We defined the full retinal volume as the volume of retinal tissue measured from the ILM to the RPE hyper-reflective line within a central 5-mm × 5-mm square. We divided the full retinal volume into an inner retinal volume and an outer retinal volume based on the outer boundaries of the inner plexiform layer.

### Statistical analysis

Because parameters may exhibit inter-eye associations within single patients, we analyzed only one eye from each patient. The statistical significance of between-group differences was determined using the Wilcoxon-rank sum test (for continuous variables) or chi-square test (for categorical variables). Spearman correlation coefficients were calculated for between-variable correlations in the RS group. All statistical analyses were performed using SPSS version 20.0 software (IBM Corp., Armonk, NY, USA). All tests were two-sided. A *p*-value < 0.05 was considered statistically significant.

## Results

Table 1 summarizes the key characteristics of the RS group and the control group. There was no significant difference in age, spherical equivalence, or axial length between the RS and control groups. The mean BCVA was 0.4 logMAR units in the RS group and 0.16 logMAR units in the control group (*p* < 0.01).

**Table 1:** Patient characteristics

	Control group	RS group	*p*-value
Patients, n	27	27	
Age, years (mean ± SD)	51.63 ± 14.58	57.19 ± 9.56	0.16
Male sex, *n* (%)	9 (33.3%)	4 (14.8%)	0.11
Spherical equivalent, diopters	−11.72 ± 4.19	−12.09 ± 6.45	0.65
Axial length, mm	28.62 ± 2.04	29.41 ± 1.54	0.14
logMAR BCVA	0.16 ± 0.19	0.4 ± 0.33	<0.01

*BCVA* best-corrected visual acuity, *logMAR* logarithm of the *minimum angle of* resolution, *RS* retinoschisis, *SD* standard deviation

**Fig. 1:**
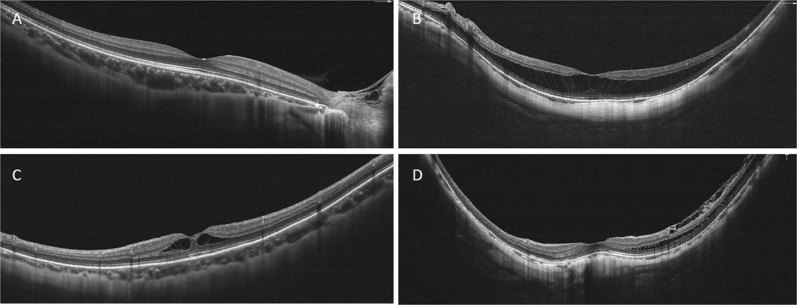
We measured subfoveal choroidal thickness and detected DSM with 10-mm cross line scans. **a**. A choroidal thickness of 215μm was measured in a myopic eye without MTM. The axial length was 27.27 mm. **b**. A choroidal thickness of 52μm was measured in an eye with retinoschisis and an axial length of 28.04 mm. **c**, **d**. Eyes with MTM without (C) and with (D) DSM had choroidal thickness values of 170μm and 83μm, respectively, and axial lengths of 28.42 mm and 31.64 mm, respectively. Abbreviations: DSM, dome-shaped macula; MTM, myopic traction maculopathy

**Fig. 2:**
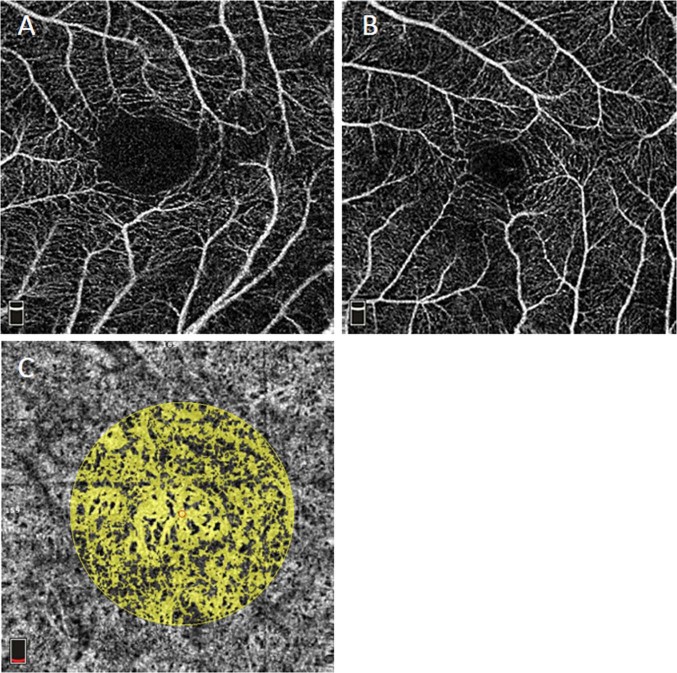
OCTA images in the SL of the retina and choroidal layers. **a**. An eye with retinoschisis in a 66-year-old woman. The area of the FAZ in the SL is 0.56mm^2^. **b**. A myopic eye without MTM in a 31-year-old woman. The area of the FAZ in the SL is 0.14mm^2^. **c**. An eye with MTM in a 45-year-old woman. The area of choroidal flow was measured by calculating the yellow-tinged area, which indicates blood flow, within a central 2-mm-diameter circle. The choroidal flow area is 2.006mm^2^. FAZ, foveal avascular zone; MTM, myopic traction maculopathy; OCTA, optical coherence tomography angiography; SL, superficial layer

**Fig. 3:**
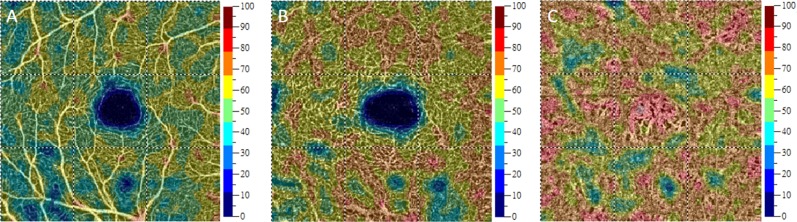
Vessel density measurements in the SL, DL, and choroid capillary layer (from left to right) in an eye with MTM. The color overlays on the OCTA images indicate the vessel density values shown in the key to the right. Whole-macula and foveal vessel density values indicate the vessel density within 3-mm × 3-mm squares and 1-mm-diameter circles, respectively. The whole-macula vessel density values in this eye were 51.81% in the SL (**a**), 60.39% in the DL (**b**), and 67.96% in the choroid capillary layer (**c**). DL, deep layer; MTM, myopic traction maculopathy; OCTA, optical coherence tomography angiography; SL, superficial layer

Table 2 presents the mean measurements for OCTA-identified biomarkers in the study groups. There was no significant difference in the signal strength index between the groups. Compared to the control group, the RS group had a significantly lower subfoveal choroidal thickness (*p* = 0.03; Fig. 1a, b), a significantly larger full retinal volume (*p* < 0.01), and a significantly larger outer retinal volume (*p* < 0.01); there was no significant between-group difference in inner retinal volume (*p* = 0.98). The RS group had a significantly larger FAZ in the SL (*p* = 0.02) and a significantly smaller FAZ in the DL (*p* = 0.02) when compared to the respective values in the control group (Fig. 2a, b). There was no significant between-group difference in choroidal flow areas (*p* = 0.32; Fig. 2c); however, the vessel density in the subfoveal choroid capillary layer was greater in the RS group (*p* = 0.04; Fig. 3). The ellipsoid zone was disrupted in two patients in the RS subgroup without DSM. All ellipsoid zones were intact in the control group.

**Table 2:** Biomarkers identified on OCTA

	Control group	RS group	*p*-value
Signal strength index	58.22	57.37	0.66
Choroidal thickness, μm	116.78 ± 71.42	80.78 ± 41.65	0.03
Retinal volume, mm^3^			
Full	6.89 ± 0.36	8.5 ± 1.97	<0.01
Inner	2.62 ± 0.4	2.6 ± 0.33	0.98
Outer	4.27 ± 0.38	5.91 ± 1.76	<0.01
FAZ-SL, mm^2^	0.27 ± 0.08	0.34 ± 0.13	0.02
FAZ-DL, mm^2^	0.73 ± 0.18	0.62 ± 0.24	0.02
Choroidal flow area, mm^2^	1.57 ± 0.47	1.46 ± 0.42	0.32
Vessel density (%)			
Whole CC	65.87 ± 2.81	63.17 ± 13.07	0.79
Foveal CC	66.03 ± 9.94	69.75 ± 6.52	0.04
Whole SL	48.12 ± 4.98	46.28 ± 6.66	0.48
Foveal SL	30.63 ± 4.47	29.69 ± 5.68	0.26
Whole DL	55.51 ± 3.98	54.82 ± 4.81	0.24
Foveal DL	30.63 ± 4.47	29.69 ± 5.68	0.26
Intact ellipsoid zone, n	27	25	0.16

*CC* choriocapillaris, *FAZ-DL* foveal avascular zone area in the deep layer, *FAZ-SL* foveal avascular zone area in the superficial layer, *n* number, *OCTA* optical coherence tomography angiography

In the RS group, DSM was present in 10 patients and absent in 17 (Fig. 1c, d). Compared to patients without DSM, those with DSM tended to be older (*p* = 0.06), and had a significantly greater axial length (30.29 mm [1.37] vs 28.89 mm [1.42]; *p* = 0.03) and a significantly smaller inner retinal volume (2.44 mm^3^ [0.3] vs. 2.68 mm^3^ [0.32]; *p* = 0.05). There was no significant between-group difference in BCVA, full retinal volume, or outer retinal volume. Compared to the patients without DSM, those with DSM had significantly lower choroidal thickness (*p* = 0.03) and a significantly greater vessel density in the choroid capillary layer in the whole-macula (*p* = 0.005) and foveal circle (*p* = 0.03).

We detected several significant correlations in the RS group. We found positive correlations between the BCVA scores for the full (*p* < 0.001), inner (p = 0.05), and outer (*p* < 0.001) retinal volumes and a negative correlation between the BCVA score and whole vessel density in the choroid capillary layer (*p* = 0.005). Axial length correlated positively with whole vessel density in the choroid capillary layer and negatively with the inner retinal volume. The area of the FAZ correlated negatively with foveal vessel density in both the SL (*p* < 0.001) and the DL (*p* = 0.02). Furthermore, the area of the FAZ in the DL correlated negatively with the foveal vessel density in the SL (*p* = 0.04). The vessel density in the SL correlated positively with that in the DL regardless of whether the whole macula (*p* = 0.007) or foveal circle (*p* = 0.001) vessel density was examined. Within the choroid capillary layer, the vessel density in the whole macula correlated positively with that in the foveal circle (*p* = 0.001). The volume of the inner retina correlated positively with the whole-macula vessel density in the SL (*p* = 0.02). Patient age was negatively correlated with foveal vessel density in the DL and positively correlated with the outer retinal volume. Full, inner, and outer retinal volumes were positively correlated with each other.

## Discussion

This study is the first to systematically evaluate MTM using OCTA to describe the severity of retinoschisis using three-dimensional analysis and to identify biomarkers on OCTA that are pathognomonic of MTM.

### Disoriented foveal Müller cell fibrils cause visual impairment

The reduced visual acuity associated with MTM of the macular hole and foveal detachment subtypes is thought to be the result of photoreceptor damage. Our findings show that the visual acuity impairments in the retinoschisis subtype result from foveal distortion rather than disruption of photoreceptors, as indicated by the increased full and outer retinal volumes and the intact ellipsoid zone in the RS group. This mechanism is very different from that found in the foveal detachment and macular hole subtypes. Although disturbances of the ellipsoid zone were found in two patients in the RS group, the logMAR BCVA scores in these patients were better than the mean value in the RS group (0.2 and 0.3 vs. 0.4), possibly because they had healthy full retinal volumes that were smaller than the mean value in this group (5.63 mm^3^ and 8.22 mm^3^ vs. 8.5 mm^3^). The significant correlation between the BCVA score and retinal volume in the RS group is consistent with the foveal distortion hypothesis, i.e., an increased retinal volume indicates more severe retinoschisis, which would aggravate foveal distortion and cause visual deterioration. Gaucher et al. did not find a correlation between central foveal thickness and visual acuity in patients with myopic foveoschisis [10]. However, the central foveal thickness is a linear measurement that cannot adequately represent the three-dimensional concept of retinoschisis cavities or the severity of foveal distortion.

Müller cells maintain the integrity of the retina by acting as optical fibers that guide light through the inner retina and minimize light-scattering. Therefore, they can enhance the signal-to-noise ratio [19]. As optical fibers, their orientation is crucial to their function [20]. The integrity of foveal Müller cells is associated with the overall structural integrity of the fovea [21]. Structural changes in Müller cells are thought to occur in eyes with retinoschisis [22]. As retinoschisis cavities enlarge, mechanical stretch forces may damage Müller cells and affect their function [23]. Therefore, we suggest that dysfunction of Müller cells leads to visual impairment in eyes with retinoschisis-type MTM.

### Choroidal perfusion is critical to the health of Müller cells and maintenance of retinoschisis in eyes with MTM

Decreased choroidal thickness is a general finding in eyes with advanced pathologic myopia [24, 25]. The decreased subfoveal choroidal thickness observed in retinoschisis-type MTM may precede the development of retinoschisis. In addition to inner mechanical stretch forces, the etiology of retinoschisis may include poor choroidal support for the health of Müller cells and consequent development of elongated fibrils on the cells. This mechanism would explain the decreased visual acuity in myopic eyes with less choroidal thickness, healthy photoreceptors, and an intact ellipsoid zone [26]. In our study, there was a positive correlation between visual acuity and whole-macula vessel density in the choroid capillary layer in the eyes with RS, which indicates the importance of choroidal perfusion. Otherwise, we found no correlation between choroidal thickness and any other study variable, including visual acuity, in the RS group. This suggests that choroidal thickness itself may not directly affect vision.

Previous reports [26, 27] noted decreased subfoveal choroidal thickness, older patient age, and an elongated axis as features that worsened visual acuity in the advanced stages of high myopia. Similarly, we found that MTM with DSM was associated with older age, a longer axis, and a thinner choroid relative to MTM without DSM. However, there was no significant difference in visual acuity between the DSM and non-DSM groups in our study. Our present findings suggest two factors that may protect against vision loss in patients with DSM. One is the buckling effect in eyes with DSM, which is reflected in smaller inner retinal volumes relative to eyes without DSM and has been mentioned in previous reports [28, 29]. The other is choroidal perfusion support, which is indicated by increased vessel density in the choroid capillary layer and may help to maintain the retinoschisis subtype and prevent detachment of the fovea. Al-Sheikh et al. proposed that decreased choroidal perfusion in myopic eyes may result from mechanical stretch forces induced by elongation of the eyeball, which DSM can relieve [30]. Higher vessel density in the choroid capillary layer of eyes with DSM may result from the buckling effect of the thickened sclera [11]. The positive correlation between whole-macula vessel density in the choroid capillary layer and visual acuity observed in the RS group indicates the importance of choroidal support. Furthermore, the observed correlations of a longer axis with a smaller inner retinal volume and higher vessel density in the choroid capillary layer in the RS group may be attributable to inclusion of DSM cases.

### The area of the FAZ is influenced by both retinal perfusion demands and mechanical stretch forces in eyes with MTM

The FAZ is known to be enlarged in the elderly and in patients with diabetes [21, 31]. However, no previous study has evaluated the FAZ in eyes with MTM. We found significant enlargement of the FAZ in the SL and a reduction of the FAZ in the DL. Samara et al. (2015) proposed that the negative correlation between the size of the FAZ and the central macular thickness in normal eyes suggests that a thicker retina has greater metabolic requirements [32]. In patients with diabetes, the FAZ enlarges in both the SL and the DL [33]. The area of the FAZ seems to reflect the perfusion status of the retina; the discrepancy that we observed between changes in the FAZ in the SL and those in the DL may be related to mechanical stretch forces rather than perfusion demands. Forces tangential to the ILM may cause enlargement of the FAZ in the SL. The staphyloma and progressive scleral stretching pull the deeper foveal tissues posteriorly, thereby narrowing the FAZ in the DL. Further structural evaluations of the foveal contours in eyes with retinoschisis are needed to test this hypothesis. We found negative correlations between FAZ areas in the SL and DL and same-layer foveal vessel density values in the RS group. There was also a negative correlation between FAZ area in the DL and foveal vessel density in the SL in the RS group. Tangential forces in eyes with retinoschisis may cause enlargement of the FAZ in both the SL and DL. Once the FAZ in the DL is enlarged, the retinal perfusion status in the SL would also be affected, thereby decreasing the vessel density. The positive correlations between vessel density in the SL and that in the DL in the RS group suggest that synchronous changes occur in both retinal layers. Furthermore, the positive correlation between the inner retinal volume and whole-macula vessel density in the SL in the RS group is consistent with the findings of previous studies in eyes without MTM [34, 35]. This corroborates our hypothesis that a thicker retina has greater metabolic demands.

In our RS group, the outer retinal volume increased with patient age, possibly in response to the aging process as well as degeneration of Müller cells and elongation of their fibrils. The retinal vessel density decreases with age in normal eyes [31]. We also found a negative correlation between patient age and foveal vessel density in the DL in our RS group.

### Study implications

In terms of surgical management, our results indicate that reversing the abnormal architecture of the Müller cell fibrils and thereby flattening the retinoschisis is the key to restoring visual acuity in eyes with MTM. Moreover, the ILM over the foveola should be preserved during ILM peeling because the foveolar ILM is part of the foveolar Müller cell fibrils. Therefore, preservation of the ILM in this region may prevent degeneration of the foveola [36]. Furthermore, choroidal perfusion, as reflected in vessel density, in eyes that have MTM without DSM should not be further impaired during surgical interventions such as external macular buckling surgery, which can further compress the choroid and decrease choroidal perfusion. Finally, distortion of the FAZ, which we found to contribute to decreased vessel density, retinoschisis, and subsequent impairment of visual acuity, should be corrected by removing the mechanical stretching forces, possibly by peeling off the epiretinal membrane, posterior hyaloid, and parafoveolar ILM.

### Limitations

Although our study provides an insight into retinoschisis-type MTM, it has several limitations. First, the sample size was relatively small. Second, OCTA has some disadvantages in that the auto-segmentation adjustments on OCTA images were performed manually rather than with a standardized automated system. Segmentation is critical for serial data analysis, especially in eyes with MTM, where every b-scan should be carefully examined. However, no software is currently available to correct segmentation errors in eyes with MTM [37]. The quality of OCTA may be influenced by many factors [38] and poor image quality might have compromised our biomarker data [39, 40]. For eyes with a high refractive error and pathology such as MTM, limiting the influence of artifacts will be important in future investigations.

## Conclusions

Our results may explain the reason why visual acuity cannot be corrected fully in some highly myopic eyes. The foveoschisis of the sensory retina may be responsible for the visual losses in myopic traction maculopathy eyes without obvious anatomical abnormalities. This condition is frequently overlooked in the refraction correction in daily practice.

Our OCTA images provided high-resolution structural and perfusion data for eyes with MTM. The morphologic and vascular perfusion measurements obtained correlated well with functional changes, which may elucidate the mechanisms underlying MTM. We found that an increased retinal volume and a distorted FAZ, which may aggravate disorientation of Müller cell fibers, were associated with greater visual impairment. It is possible that compensatory increased perfusion, as indicated by increased vessel density, may have a rescue effect in eyes with DSM and retinoschisis and prevent development of foveal detachment or macular holes. Further investigations, including more histopathologic studies, are necessary to confirm the pathogenesis of MTM. Such studies may elucidate the role of Müller cell integrity in traction maculopathies because MTM is a pathology of Müller cell cones that involves both inner traction from the ILM and outer stretch forces from posterior staphyloma.

### Summary

#### What was known before

- In some highly myopic eyes with myopic traction maculopathy the visual acuity cannot be well-corrected. The mechanism is not fully understood in these eyes with macular retinoschisis.

#### What this study adds

- This study resolves the mechanism underlying an overlooked entity that exists in highly myopic eyes, which hinders the best refraction correction in a seemly normal retina. The functional and anatomical correlation may be connected in myopic traction maculopathy by optical coherence tomography.

## References

[1] Takano M, Kishi S (1999). Foveal retinoschisis and retinal detachment in severely myopic eyes with posterior staphyloma. Am J Ophthalmol.

[2] Panozzo G, Mercanti A (2004). Optical coherence tomography findings in myopic traction maculopathy. Arch Ophthalmol.

[3] Foster PJ, Jiang Y (2014). Epidemiology of myopia. Eye.

[4] Wong YL, Saw SM (2016). Epidemiology of pathologic myopia in Asia and worldwide. Asia Pac J Ophthalmol.

[5] Ikuno Y, Sayanagi K, Soga K, Oshima Y, Ohji M, Tano Y (2008). Foveal anatomical status and surgical results in vitrectomy for myopic foveoschisis. Jpn J Ophthalmol.

[6] Taniuchi S, Hirakata A, Itoh Y, Hirota K, Inoue M (2013). Vitrectomy with or without internal limiting membrane peeling for each stage of myopic traction maculopathy. Retina.

[7] Benhamou N, Massin P, Haouchine B, Erginay A, Gaudric A (2002). Macular retinoschisis in highly myopic eyes. Am J Ophthalmol.

[8] Gaucher D, Haouchine B, Tadayoni R, Massin P, Erginay A, Benhamou N (2007). Long-term follow-up of high myopic foveoschisis: natural course and surgical outcome. Am J Ophthalmol.

[9] Shimada N, Tanaka Y, Tokoro T, Ohno-Matsui K (2013). Natural course of myopic traction maculopathy and factors associated with progression or resolusion. Am J Ophthalmol.

[10] Gaucher D, Erginay A, Lecleire-Collet A, Haouchine B, Puech M, Cohen SY (2008). Dome-shaped macula in eyes with myopic posterior staphyloma. Am J Ophthalmol.

[11] Ellabban AA, Tsujikawa A, Matsumoto A, Yamashiro K, Oishi A, Ooto S (2013). Three-dimensional tomographic features of dome-shaped macula by swept-source optical coherence tomography. Am J Ophthalmol.

[12] Freiberg FJ, Pfau M, Wons J, Wirth MA, Becker MD, Michels S (2016). Optical coherence tomography angiography of the foveal avascular zone in diabetic retinopathy. Graefes Arch Clin And Exp Ophthalmol.

[13] Kim JY, Kwon OW, Oh HS, Kim SH, You YS (2016). Optical coherence tomography angiography in patients with polypoidal choroidal vasculopathy. Graefes Arch Clin Exp Ophthalmol.

[14] Yoshida-Uemur T, Katagiri S, Yokoi T, Nishina S, Azuma N (2017). Different foveal schisis patterns in each retinal layer in eyes with hereditary juvenile retinoschisis evaluated by en-face optical coherence tomography. Graefes Arch Clin Exp Ophthalmol.

[15] Gao SS, Jia Y, Zhang M, Su JP, Liu G, Hwang TS (2016). Optical coherence tomography angiography. Invest Ophthalmol Vis Sci.

[16] Spaide RF, Koizumi H, Pozzoni MC (2008). Enhanced depth imaging spectral-domain optical coherence tomography. Am J Ophthalmol.

[17] Klufas MA, Phasukkijwatana N, Iafe NA (2017). Optical coherence tomography angiography reveals choriocapillaris flow reduction in placoid chorioretinitis. Ophthalmoly Retina.

[18] Kuehlewein L, Bansal M, Lenis TL, Iafe NA, Sadda SR, Bonini Filho MA (2015). Optical coherence tomography angiography of type 1 neovascularization in age-related macular degeneration. Am J Ophthalmol.

[19] Reichenbach A, Bringmann A (2015). New functions of Müller cells. Glia.

[20] Franze K, Grosche J, Skatchkov SN, Schinkinger S, Foja C, Schild D (2007). Müller cells are living optical fibers in the vertebrate retina. Proc Natl Acad Sci USA.

[21] Gass JD (1999). Müller cell cone, an overlooked part of the anatomy of the fovea centralis: hypotheses concerning its role in the pathogenesis of macular hole and foveomacular retinoschisis. Arch Ophthalmol.

[22] Tang Johnny, Rivers Michael B., Moshfeghi Andrew A., Flynn Harry W., Chan Chi-Chao (2010). Pathology of Macular Foveoschisis Associated with Degenerative Myopia. Journal of Ophthalmology.

[23] Park S, Lee YJ (2013). Nano-mechanical compliance of Müller cells investigated by atomic force microscopy. Int J Biol Sci.

[24] Fujiwara T, Imamura Y, Margolis R, Slakter JS, Spaide RF (2009). Enhanced depth imaging optical coherence tomography of the choroid in highly myopic eyes. Am J Ophthalmol.

[25] F lores-Moreno I, Lugo F, Duker JS, Ruiz-Moreno JM (2013). The relationship between axial length and choroidal thickness in eyes with high myopia. Am J Ophthalmol.

[26] Nishida Y, Fujiwara T, Imamura Y, Lima LH, Kurosaka D, Spaide RF (2012). Choroidal thickness and visual acuity in highly myopic eyes. Retina.

[27] Gozum N, Cakir M, Gucukoglu A, Sezen F (1997). Relationship between retinal lesions and axial length, age and sex in high myopia. Eur J Ophthalmol.

[28] Baba T, Tanaka S, Maesawa A, Teramatsu T, Noda Y, Yamamoto S (2006). Scleral buckling with macular plombe for eyes with myopic macular retinoschisis and retinal detachment without macular hole. Am J Ophthalmol.

[29] Mateo C, Bures-Jelstrup A, Navarro R, Corcostegui B (2012). Macular buckling for eyes with myopic foveoschisis secondary to posterior staphyloma. Retina.

[30] Al-Sheikh M, Phasukkijwatana N, Dolz-Marco R, Rahimi M, Iafe NA, Freund KB (2017). Quantitative OCT angiography of the retinal microvasculature and the choriocapillaris in myopic eyes. Invest Ophthalmol Vis Sci.

[31] Iafe NA, Phasukkijwatana N, Chen X, Sarraf D (2016). Retinal capillary density and foveal avascular zone area are age-dependent: quantitative analysis using optical coherence tomography angiography. Invest Ophthalmol Vis Sci.

[32] Samara WA, Say EA, Khoo CT, Higgins TP, Magrath G, Ferenczy S (2015). Correlation of foveal avascular zone size with foveal morphology in normal eyes using optical coherence tomography angiography. Retina.

[33] Takase N, Nozaki M, Kato A, Ozeki H, Yoshida M, Ogura Y (2015). Enlargement of foveal avascular zone in diabetic eyes evaluated by en face optical coherence tomography angiography. Retina.

[34] Yu J, Gu R, Zong Y, Xu H, Wang X, Sun X (2016). Relationship between retinal perfusion and retinal thickness in healthy subjects: an optical coherence tomography angiography study. Invest OphthalmolVisl Sci.

[35] Landa G, Garcia PM, Rosen RB (2009). Correlation between retina blood flow velocity assessed by retinal function imager and retina thickness estimated by scanning laser ophthalmoscopy/optical coherence tomography. Ophthalmologica.

[36] Ho TC, Yang CM, Huang JS, Yang CH, Yeh PT, Chen TC (2014). Long-term outcome of foveolar internal limiting membrane nonpeeling for myopic traction maculopathy. Retina.

[37] Eladawi N, Elmogy M, Helmy O, Aboelfetouh A, Riad A, Sandhu H (2017). Automatic blood vessels segmentation based on different retinal maps from OCTA scans. Comput Biol Med.

[38] Spaide RF, Fujimoto JG, Waheed NK (2015). Image artifacts in optical coherence tomography angiography. Retina.

[39] Al-Sheikh M, Falavarjani KG, Akil H, Sadda SR (2017). Impact of image quality on OCT angiography based quantitative measurements. Int J Retina Vitreous.

[40] Cheung CMG, Arnold JJ, Holz FG, Park KH, Lai TYY, Larsen M (2017). Myopic choroidal neovascularization: review, guidance, and consensus statement on management. Ophthalmology.

